# Improving the Practice of Prosthetic Dentistry

**Published:** 1921-07

**Authors:** W. F. Lasby


### IMPROVING THE PRACTICE OF PROSTHETIC DENTISTRY

BY W. F. LASBY, D.D.S.

This paper deals with the question of the dental colleges improving the practice of prosthetic dentistry, by teaching the subject in an adequate manner to students —giving courses for the training of mechanic assistants for dental offices, and also by offering extension courses to dentists who desire further instruction so that they may be better qualified to render the service expected of them by their clientele.

A change has been taking place in the practice of dentistry. Available data, although somewhat incomplete, tend to show that in the past three years there has been an increase of approximately 80 per cent. in the demand for full dentures and more than 100 per cent. in the demand for partial denture restorations. This is indicative of the increased time and attention required of the dentist in this branch of his practice and of its increased importance to the whole profession. Much of this increased demand, it must be noted, is not due to neglect or carelessness on the part of our patients, but it is due to the failure of the restorative work which we have done for them, which has proved to be unsound in principle, because of the pathological conditions developed, and mechanically defective in execution. These same patients, with mouths partly or wholly edentulous, are again appealing to us for relief from the unfortunate condition in which they find themselves and for which we are in part responsible. Furthermore, it seems very probable that a period of from five to ten years must pass before this wave of increased denture demand due to the removal of foci of infection from the mouth subsides and we return to the normal increase in the demand for this service. This situation should arouse in us a desire to make a thorough study of the shortcomings of denture service as now practiced and stimulate us to find a solution to the difficulties with which we are meeting. Here and there we find men who are serving their clientele well and we all may at times feel a bit of justified pride in the results we are geeting. Yet it is evident that as we talk with dentists we find that they seem more concerned about the success or failure of this branch of their work than they formerly were, and some of them are looking forward to the time when there will be no more dentures to be made or when there will be some one else in their community who will do the work for them. Then, too, the patients are better informed in regard to what can be done for an edentulous mouth and are more exacting in their demands for artistic and efficient restorations.

With these considerations before us, then—the increased demand for dentures, the better informed and more exacting patients seeking our service, and our responsibility toward them as a profession—to whom shall we look for help in trying to improve our denture work if not to the colleges? They are the already established agency in every section of the country for the teaching of dentistry and for research work in the problems that come up for solution. If these colleges are to prove true to the purpose for which they exist they will prove equal to the task imposed upon them in this work and the results will justify our confidence in them. It is around these colleges that we believe the leaders of this work should rally and carry forward the educational work in an effective manner for both students and dentists.

Our educational problem is to adjust the courses of study to meet the constantly changing requirements of practice. The colleges have advanced from a three to a four-year course. The five-year course is already announced by one university. The probability is that we shall become a specialty of medicine and have a four-year course leading to the degree of bachelor of medicine and follow it with a course of two or three years to prepare for any one of the specialties of medicine, including that of dentistry. It is during this period of rapid transition that we should especially seek to preserve a rational curriculum in dental subjects. Let us see to it that sufficient time and training is given in technical branches to develop in the student that degree of skill and judgment so essential in the execution of restorative and replacement work in the mouth. More skill is now required of the dentist in his operations on vital teeth to preserve them, to maintain the soft tissues of the oral cavity in a healthy condition, and to safeguard the patients' health than we formerly required. Therefore in planning a rational course of study we must not forget that as we lengthen the years of study sufficient time must be allotted to the training of the eye and hand and not permit all the additional time to be used in the theoretical subjects of the course.

What shall we expect of the colleges in the teaching of prosthetics to students? The first requirement is that they shall provide a sufficient number of trained teachers to give personal instruction to every student in the class. A lecturer may reach the ears of a large class and hold their attention in covering the didactic phases of the work; a demonstrator may show a fairly large group how a certain piece of technic should be done; but when it comes to the actual doing of the work in an intelligent way as regards detail and perfection, every student requires personal instruction of an intensive type. Such attention can be given by a teacher to a group of from ten to fifteen in technic courses and about eight in the clinical work. There is no substitute for the active, enthusiastic, effective teacher who gives to every student the help needed without delay in the work of the college. Not all colleges are so provided with teachers. Some expect an instructor to teach prosthetic technic to a class of fifty, or seventy-five, or even more, and it is absurd to expect that it can be done in any such manner. Every student should receive personal attention and we as a profession should require our colleges to provide a sufficiently large number of trained teachers to meet the requirements of the students in this respect.

The next requirement is for fully equipped laboratories with individual bench units, well lighted and ventilated and kept in a thoroughly clean and sanitary condition. No teacher can permanently overcome the handicap of a poorly lighted, dirty, untidy room which breaks down the morale of the class and fails to stimulate the students to do their best work. The modern laboratory should be comparable to the modern operating room, thoroughly prepared for the purpose it is to serve and a stimulus to every one to do his best work.

After the question of teachers and laboratories comes that of what constitutes a course in prosthetic technic. The keynote should be adequate training for present-day methods of practice and the abandonment of old or obsolete pieces of technic and methods. Our present need in this work is a standardized technic covering all phases of the work. It should be complete, comprehensive, and yet simplified so that in a busy office all details aside from the actual operations at the chair can be delegated to an assistant of moderate training and intelligence. It is vitally essential in the conduct of a practice that production be upon a sound basis financially. Prosthetic dentistry in many offices is done at a loss because the dentist himself does not know the cost of producing a denture; he makes the mistake of contracting to give a patient a satisfactory result without a thorough knowledge of the mental condition of the patient, his attitude toward the work to be done, and an understanding of the mouth conditions involved. When we add to this a haphazard slipshod technic of denture construction made by the dentist, by his assistant, or by a commercial laboratory, we have a combination of possibilities for professional and financial failure. The solution can come only from the educational side. The dentist must be prepared while in college to thoroughly understand and diagnose mouth conditions for denture work and have a standardized, simplified, accurate technic to be followed by himself and assistants in order to put production on the correct side of his ledger account.

In addition to lectures and demonstrations and study of the properties of the materials used in the work, it is our teaching practice to have in laboratory pieces of technic work showing in detail every step of the work required, from the beginning to the finished product, so that the student has a visualized and tangible idea of what he is to do. As each step of the work is completed it is inspected and the student's card checked by the instructor. We know how fast each student is progressing, that he is doing the work himself, and that he understands what he is doing. Mistakes are easily discovered and corrected, and a wholesome rivalry is developed in the class as the students note the progress made by each other. As the finished work is placed on exhibition before the whole class no student willingly wants a defective piece to be seen by his classmates.

The training of mechanical assistants for dental office laboratories should include the same technic as that given to dental students and where adequate teaching instruction is given, one year is found to be sufficient to cover the work in all technic branches required to prepare them for office positions.

As a profession, we have technic systems with names attached, we have systems without names or where names have been forgotten, and we have methods without number, all have merit, some are good and some not so good. All are short of the ideal. What the colleges need to teach at the present time is not methods and systems, but a standardized technic, founded on a thorough knowledge of the tissues of the oral cavity involved and the physical properties of the materials used in the construction of the denture. Why should this not be done? Human mouths present very much the same conditions in all parts of the world where dentistry is practiced and there is no reason why a technic used in one locality is not applicable to any other. Cavities to be filled with gold foil vary in size and form and yet the operation is more successful when the work done conforms to established procedures. In like manner gold inlay cavities are different, but the inlay fits the cavity better when a standardized technic of making the wax model, investing, burning out, and casting the inlay is followed than when a haphazard method is used. Likewise a standardized prosthetic technic gives better results. We would have had it long ago if we could have agreed on details. We overemphasize certain details and forget to build a complete structure in our enthusiasm over some newly discovered angle of the problem.

We are glad of the progress recently made to produce an articulator for universal use. The selfish hope of gain and fame on the part of men who have been the leaders in the work has delayed the development of the instrument. The movements of the human mandible do not present different aspects in different locations; thus our problem is not one of meeting local conditions, but the bringing together of localized methods and systems and the establishment of a standardized technic. Colleges may then teach it to qualified students of dentistry in suitably equipped laboratories, and after their graduation the profession will be honored by receiving into its ranks men who will be a credit to the healing art of dentistry.

Why should the colleges of the country not teach denture prosthesis on such a basis? The need is apparent to every one who has studied the situation as it exists at the present time. As a profession we have a right to expect it. If any colleges fail to make progress it is because their vision of the educational ideal is eclipsed by the hope of financial gain, which they have before them as the reason for their existence. The most unfortunate feature of dental educational work in this country to-day is that there are institutions where financial returns to the owners of the enterprise seem to be the chief reason for their continuation. Except for a few headliners their faculties are hopelessly inadequate to do effective teaching, their classrooms and laboratories overcrowded and poorly equipped, and the whole situation depressing to those who are vitally interested in the future progress of our work. We should be profoundly grateful for the survey of the war educational board which classified the colleges of dentistry for us and called attention to the conditions under which some of them were existing. Dental education should be handled without thought of financial gain to any institution and can be adequately taught to meet present-day requirements. only in colleges fully equipped and supported by outside financial aid.

The other phase of our problem is to aid the dentist already in practice and offer him the opportunity to keep up with the progress of the profession by means of intensive courses of study in any branch which he may choose to take. This opens the subject of extension courses and it is here that the colleges have an opportunity and an obligation to fulfill in their educational work. Some have vitally interested themselves in the problem and have made a creditable beginning and we bespeak for their honest endeavors the whole-hearted support of the profession.

We are familiar with the attractive announcements so frequently received of courses to be given by individuals or societies purporting to give instruction in almost any branch of dentistry. We wish that we might bespeak for such courses our approval, but we can not do so. The earnest, studious, hard-working, conscientious dentist, with high ideals of service for his clientele, who seeks to improve himself, gains from such courses an inspiration to perfect his technic and returns a better student and practitioner. For this type of dentist we can see some justification for such courses. What about another type? We regret to say that they exist and that there are more of them than there should be. We refer to those whose aim in the practice of dentistry is primarily to get the most income for the least effort and who sell their product at all times with that end in view. Can you become a specialist in local anesthesia and dental surgery by watching some one else do that kind of work for a few days? And yet you know men who are posing as specialists in oral surgery after just such training. The public and the true advancement of dentistry is suffering in consequence of such methods.

A thorough course in the theory and practice of any specialty is the only type of post-graduate or extension course of study which should receive the approval or sanction of any college or dental society. No other satisfactory pedogogical method has ever been devised to meet the needs of the case. Dentists may be enthusiastic and stimulated by other methods, but not educated and trained to properly qualify them for the work they may wish to do.

The University of Minnesota has for some time admitted graduates who wished to pursue studies in any branch of dentistry, but no certificate of any kind is granted except as the work could be applied toward a degree in the graduate school. Extension courses are offered to classes at stated intervals and the work has been well received. Three prosthetic courses given under the direction of Dr. M. M. House, of Indianapolis, Indiana, have shown that men are waiting for the opportunity to do some real work in denture prosthesis. We find that four weeks is required for dentists taking their first course. A staff of one director and four assistants is required for a class of twenty-four in order to teach the work in a satisfactory manner. The first week is devoted to lectures and demonstrations. The second week the men go into the laboratory and construct a technic case carrying out in detail, under careful instruction, the accurate and scientific technic shown in lectures and demonstrations. During the third and fourth weeks the practical work is done for patients, ample time being used so that each one studies and observes the various and difficult conditions present in the mouths of all the patients. After having had two courses of four weeks each, 43 per cent. of the men registered for a two weeks' continuation course. The work is just getting started and presents almost unlimited possibilities. Most of the men taking the courses thus far are general practitioners and not prosthetic specialists. They represented nineteen states and the Dominion of Canada. It does not seem to be a question of location, time or expense, but it was evident that they were earnestly seeking for a chance to improve their practice of denture prosthesis. Men of this type are to be found in every community and the opportunity for them to register for extension courses of instruction given in an adequate manner should be offered by every dental college in the country.

Fully equipped and endowed colleges of dentistry, trained teachers to give personal instruction to all students, a scientific technic of denture construction, extension courses of real value to dentists—these are the essential factors in the improvement of denture prosthesis at the present time.

